# Review of tDCS Configurations for Stimulation of the Lower-Limb Area of Motor Cortex and Cerebellum

**DOI:** 10.3390/brainsci12020248

**Published:** 2022-02-11

**Authors:** Vicente Quiles, Laura Ferrero, Eduardo Iáñez, Mario Ortiz, José M. Azorín

**Affiliations:** 1 Brain-Machine Interface System Lab, Miguel Hernández University of Elche, 03202 Elche, Spain; lferrero@umh.es (L.F.); eianez@umh.es (E.I.); mortiz@umh.es (M.O.); jm.azorin@umh.es (J.M.A.); 2 Centro de Investigación en Ingeniería de Elche-I3E, Miguel Hernández University of Elche, 03202 Elche, Spain

**Keywords:** tDCS, lower-limb stimulation, motor cortex, cerebellum

## Abstract

This article presents an exhaustive analysis of the works present in the literature pertaining to transcranial direct current stimulation(tDCS) applications. The aim of this work is to analyze the specific characteristics of lower-limb stimulation, identifying the strengths and weaknesses of these works and framing them with the current knowledge of tDCS. The ultimate goal of this work is to propose areas of improvement to create more effective stimulation therapies with less variability.

## 1. Introduction

Non-invasive brain stimulation techniques (NIBS) have been used historically to treat neuronal diseases. Recently, these techniques have gained interest, especially in the research sector. In several works, these techniques are applied with the aim of aiding in the rehabilitation process. Two of the most emergent NIBS are transcranial magnetic stimulation (TMS) and transcranial direct current stimulation (tDCS).

TMS generates a magnetic field in the brain through an insulated wire coil placed in the skull. This generates a transient current in the brain that is capable of depolarizing neurons with upper-threshold intensity. TMS combined with electromyography (EMG) allows for the recording of motor evoked potentials, which are used as an indicator of excitability. Depending on the frequency, the duration of the stimulation, and the strength of the magnetic field, TMS can activate or suppress neuronal activity [1].

tDCS is a non-invasive technique that modulates brain excitability, based on altering the resting membrane potential of neurons by means of a small direct electrical current sub-threshold [2]. Depending on the direction of the current relative to the orientation of the neuron, depolarization or hyperpolarization can occur [3]. According to the literature, neuronal excitation occurs with anodal stimulation and inhibition with cathodal stimulation [4]. There are several important parameters to achieve the desired effect, such as current density, electrode size, electrode placement and stimulation polarity.

Neuromodulation by tDCS is thought to follow Hebbian Theory (“neurons that fire together, wire together”). If the presynaptic and postsnaptic neurons are activated at the same time, there will be a strengthening of the synapse. Depending on the excitation or inhibition, this phenomenon is called long-term potentiation (LTP) or long-term depression (LTD) [5]. In addition, this enhancement can be modulated by endogenous plasticity depending on the tasks that the user is performing, according to the theory postulated by Elie Bienenstock, Leon Cooper and Paul Munro, known as BCM [6]. tDCS techniques are used in combination with other methodologies and therapies to study their benefit in modulating the mechanism of neuronal plasticity, which is very important in the motor rehabilitation of people who have suffered a stroke. Sometimes, these people have to look for alternative mechanisms that allow them to perform certain types of movement. Brain-computer interfaces (BCI) can help strengthen these connections by finding alternative ways to generate the association between thoughts and motor action [7].

tDCS, together with other stimulation techniques, is the subject of this study because of its ease of combination with BCI and its potential benefits in clinical settings [8]. The usefulness of tDCS [9] and BCI [10] has been tested separately; however, there is a lack of literature concerning the combination of both. In this paper, the key aspects for a tDCS design study are discussed based on previous lower-limb tDCS works. Although tDCS studies have high variability in the implementation parameters and the conditions under which the therapies are performed [11], it is possible to delimit the most important features.

Regarding tDCS polarization mechanisms, the orientation of the current flow with respect to the position of the neurons may play an important role. In the cerebral cortex, the most abundant neurons are pyramidal and are oriented perpendicularly. In this case, the anode on the cortex depolarizes the basal dendrites and hyperpolarizes the apical dendrites, while the cathode depolarizes the apical dendrites, producing, under certain characteristics, excitation. This way, the cathode hyperpolarizes the basal dendrites, producing, under certain characteristics, inhibition [12]. Another important key is the cerebral cortex irregularities. The field can penetrate the cortex at different angles. When stimulating the motor homunculus, which is the area related to the coordination of the different motor areas, the most difficult area to reach perpendicularly is the one related to the primary motor cortex (M1) cerebral leg area since the orientation of the neurons is parallel to the cortex surface. Another area involved in the coordination of movements is the cerebellum [13]. It is easier to stimulate, and the stimulation is usually applied 3 cm lateral to one hemisphere. Regarding the position of the cathode and anode, two types of assemblies are distinguished: cephalic monopolar and bipolar positions. In the cephalic monopolar position, the active electrode is located on the skull and the electrode of the opposite sign is located in extra cephalic.

Regarding the type of electrode, sponge electrodes with a contact area between 25 cm${}^{2}$ and 35 cm${}^{2}$ are usually used, although smaller areas have also been tested. It is recommended to not have areas below 9 cm${}^{2}$ [14]. High-definition (HD) electrodes are another variant of the tDCS electrode array; they have a skin contact area of less than 5 cm${}^{2}$, and they usually have a cylinder shape. The HD electrode includes a cylindrical base that sits on the skin and determines the contact area. The cylindrical base is filled with conductive gel or paste. Suspended above the gel, there is a ring, disk, or pellet made of Ag/AgCl.

Regarding the stimulation time in the therapeutic use of tDCS, sessions of 10–30 min are usually applied, which have short-term effects: they can produce changes that last from 30 to 120 min [15]. Additionally, if these sessions are performed repeatedly over days, the therapeutic effects could be prolonged in time, between three and six months [16,17,18].

Regarding the results of the task and the protocol, studies in which stimulation is paired with a behavioral treatment can be combined in two ways: online if stimulation is provided while the task is being performed, or offline if the task is performed after the stimulation. In some studies, tDCS is accompanied by a motor task but not by the main rehabilitation task (semi-online way). Some studies remark on the advantages of online stimulation over offline stimulation [19]. However, this can largely depend on the type of task being performed. In any case, these statements do not have enough evidence, and it is yet to be studied. The evaluation is done differently depending on what is to be tested and the task performed, strength, mobility, balance, etc. In addition, the recording of brain activity is also used as an indicator of the efficacy of tDCS. As described above, TMS is often used to measure cortical activity. In fact, it is corroborated that the use of measures such as EEG could contribute to reducing the variability of tDCS [20].

Which is the task to use in combination with tDCS for optimizing the results is an open question. Brain–machine interfaces are a natural way to establish a connection between brain activity and muscle activity; this connection may be partially weakened by a spinal cord injury or neuronal death caused by a stroke. tDCS is typically used in combination with traditional rehabilitation therapy. In this study, besides studying these types of outcomes, their combination with BCI technology was investigated. Several paradigms for rehabilitation have been tested in brain–machine interfaces, with motor imagination (MI) being one of the most common. The MI process could be described as the representation of MI in which the subject visualizes their body performing a task [21]. This paradigm is plausible to be recorded and characterized through EEG, and users can easily regulate this activity [22]. Furthermore, these cognitive processes may enhance rehabilitation through the mechanism of MI plasticity [23]. Other therapies such as robotic assistance, which has partially proven its efficacy as an improvement in rehabilitation [24,25], are easily combined with tDCS to reduce the recovery time. Robotic tools are used in the rehabilitation of the lower limb for patients with motor disabilities, instead of traditional gait-assistance methods, such as crutches, walkers or orthosis. These robotic mechanisms actively assist walking, with different degrees of assistance and independence of movement, from a lower level of independence Lokomat, to a higher degree, including ambulatory exoskeletons.

Finally, an important issue to take into account is the experimental design for evaluating the benefits of tDCS. Two methodologies have been found for hypothesis validation: one subject performs all experimental conditions, leaving a waiting time between them, or the subjects are divided into two groups for each of the experimental conditions. Depending on the type of stimulation, it is important to leave enough time between trials to avoid pooling effects. Normally, in tDCS protocols, real stimulation is used for one group of participants (sham) and simulated stimulation for the other group (control).

The aim of this study is to analyze lower-limb-focused tDCS assemblies and propose a way forward for the design of future configurations. In each section, some of the most important aspects to understand how the parameterization can affect the stimulation results have been analyzed.

## 2. Materials and Methods

To evaluate the current state of the literature, a systematic review of all original published works related to tDCS applied at the lower-limb cortex was performed, following the PRISMA guidelines [26,27].

### 2.1. Search Strategy and Article Selection

In order to search for papers that help to choose strategies in the development of a protocol for lower-limb stimulation, an extensive search was carried out in Jun 2021 through NCBI PubMed. Several conditions have been tested for papers published between 2010 and 2021: tests on stroke patients, tests on spinal cord injury patients and tests on healthy subjects. In addition, works have been included with the description tDCs, accompanied by terms such as lower limb, motor imagination or BCI. Articles were excluded if they did not provide detailed or clear information about experimental procedure performance. Besides, extra papers that were mentioned in the screening papers were added. Within the tDCS literature, papers have also been included in which the more specific effects of tDCS are discussed through computer model simulations.

### 2.2. Review Criteria

In the eligibility phase, the following inclusions criteria were checked: papers with configurations totally or partially focused on the M1 cortex related to the legs have been chosen. The paper must have some kind of control group related to the task, type of stimulation or setup. The groups of words combined with the AND conditioner were as follows:

- tDCS and stroke and gait/tDCS and stroke and lower-limb;
- tDCS and SCI and gait/tDCS and SCI and lower-limb;
- tDCS and healthy and gait/tDCS and healthy and lower-limb.

Terms such as MI and BCI have been added in order to have the necessary references for papers including brain interfaces.

- tDCS and MI;
- tDCS and BCI.

Papers have been divided according to the type of users as these papers have similar conditions and will facilitate discussion.

### 2.3. Data Extraction

The scales chosen to define the works are as follows: number of users; if they are stroke or spinal cord patients; if they were divided into experimental conditions or if they all performed all the experimental conditions; location and size of the anode and cathode; intensity and density of anode and cathode currents; number of sessions; type of task; type of analysis: online or offline; types of stimulation groups; measurement distance; time period between groups; stimulation task; types of control group; stimulation time; and evaluation scales.

## 3. Results

### 3.1. Selected Studies and Quality

**Figure 1:**
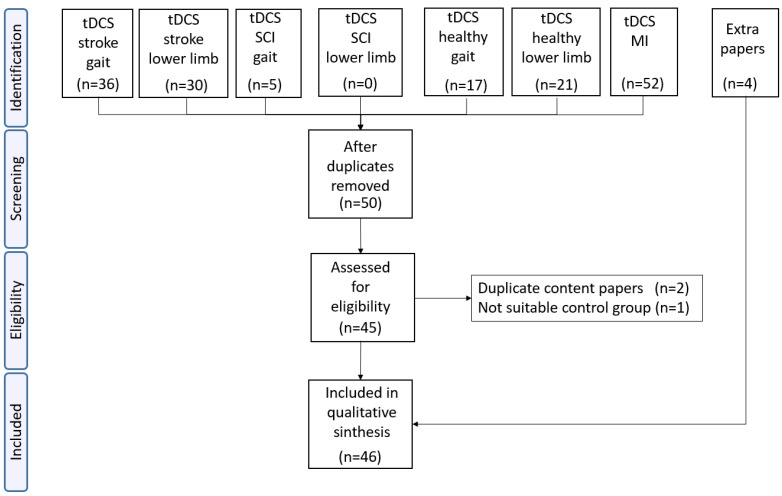
Of the 161 papers found, only 50 passed the inclusion criteria. After eliminating duplicates, only 45 papers were analyzed in the eligibility phase. Three of them were discarded due to being papers published in reduced versions or papers with control groups that did not meet the inclusion criteria. Four additional papers were added due to their relevance and because they were cited in other works. A total of 46 papers were finally included in the final analysis.

In the first phase of identification, 161 papers from each of the searched categories were included. Duplicated papers and those that did not contain the key words were eliminated (see Figure 1).

### 3.2. Difference between Healthy and Patients Study

There are parameters that are difficult to categorize but that can affect the efficacy of tDCS, for example, age, sex, type of injury, and degree of disability [28]. In this work, it has been analyzed whether the users are patients or healthy subjects. The time that has passed since the injury has been considered:

- Stroke: in the stroke studies, no distinctions have been made between ischemic or hemorrhagic. Of all the studies searched, 12 were in chronic, 6 in sub-acute and 1 in acute stage.
- Spinal cord injury: two studies in incomplete spinal cord injury, one in chronic phase and another in acute phase.

Previous studies have performed stimulation in a single session or in multiple sessions. In this study, conclusions from both types of studies have been drawn, since studying each of the parameters of the stimulation protocol is pretended. Studies in the literature that perform an analysis of some parameters in a single session cannot be underestimated. However, this difference is taken into account to positively evaluate those studies with repeated-sessions.

### 3.3. Study Designs for the Application of tDCS

Grouping by tables has reduced heterogeneity, and it allows one to better track the advances and innovations of the different works. Throughout the text, these three groups will be analyzed separately: Stroke (see, Table 1), Spinal Cord Injury (see, Table 2) and Healthy (see, Table 3). The table details some of the most relevant aspects found in the bibliography to categorize the works. These details were used to analyze the works detailed in Section 2.3.

Although this facilitates comparison, there continues to be great heterogeneity. Within these categories, there are many differences between studies: position and laterality, different electrode sizes, different types of stimulation Anodal vs. Cathodal, current strength, types of stimulation (offline vs online), number of sessions and different types of concomitant task. To reduce this variability, each of these parameters was analyzed.

### 3.4. Task Performance and Probing tDCS Effects

The effect of tDCS is usually quantified indirectly by comparing two scenarios. Researchers measure whether a certain tDCS polarity modulates a behavior that would not occur in a simulated situation. Therefore, the position of the active electrodes and the choice of the task and its associated metric are fundamental to discern the effects of tDCS.

Three measurements are usually performed to compare tDCS results: pre (before the intervention, measure used for a later comparison), during (during the main task, metrics are evaluated accordingly) and post (just after finishing the intervention or a few hours after finishing it and in the long term between 1 and 6 months after the end of the trial). As mentioned in the introduction, depending on whether tDCS is performed in a single session or in multiple sessions, the effects can be prolonged more in time and, therefore, it would make sense to monitor progress, performing these evaluations continuously.

Another topic to take into account is whether the subjects are divided into different groups to validate the research hypotheses or whether each subject belongs to several groups. If a subject performs several experimental conditions, it is recommended to leave between 48 h and 1 week [11] between sessions. However, these effects may be more or less persistent depending on the protocol and the configuration chosen. It has been proven that with a standard configuration, a distance of 7 days does not produce accumulated effects [54].

Regarding the methodologies to measure changes in tDCS, direct or indirect measurements can be chosen. Direct measurements measure or model how electrical flow crosses brain structures and polarizes neurons. In addition, it is necessary to study how the oscillations and correlations of different brain areas produce different behaviors, which are usually measured with indirect measurements. The indirect methodologies are the behavioral measures.

Starting with direct measurements, one of the measures that serves as gold-standard to determine direct relationship between tDCS and induced brain electric fields (EF) is computational modeling:

- Computational models are based on determining the influence of the EF on the tissue. Spherical models or realistic models based on MRI scans are used for this purpose.

Methodologies that can measure tDCS polarity effects:

- Evaluation by transcranial magnetic stimulation (TMS) of the cortico-motor excitability (CME) with motor evoked potentials (MEPs) recorded by electromyographic electrodes (EMG). Typical measures for quantifying MEPs are amplitude or peak to peak interval.
- Quantification of the evolution of neural activity by means of EEG recording: (i) By percentage of the correct classification distinguishing two mental task, typically MI. The signal is characterized at certain frequencies where tDCS could produce significant variation after application. (ii) Assessing connectivity between brain areas while performing an MI task. Several studies report changes in connectivity after stimulation.

The following are indirect assessment measures similar to cognitive tests, mainly physical and motor assessment tests:

- Functional assessment: scales to assess motor improvement, especially in tDCS interventions with stroke, spinal cord injury and Parkinson’s patients but also in healthy patients. Metrics such as balance, walking parameters, contraction or strength test. Many scales can measure several of these categories at the same time.
- Tests more focused on the cognitive part: symbol digit modalities test or Word Test.

The tasks performed are usually not directly linked to the point of stimulation (focused on a single muscle group). When the study includes hotspot positioning using TMS, a more direct stimulation over the target area is ensured.

### 3.5. Electrode Position and Laterality

Different electrode configurations found in the literature have been analyzed. As well as in the literature, the importance of the EF magnitude and direction on the cerebral cortex is being studied [72,73,74,75]. To understand the relevance of the position and relative distance between electrodes, the following factors must be taken into account: distance between active and return electrodes increases the magnitude of the peak EF [76,77], and the position of the return electrode even affects the current under the active electrode [78].

Among the studies describing set-ups for targeting the lower-limb-related area, there are those that attempt to target one of the two hemispheres by focusing on a particular muscle group (target leg) or those that place the active electrode in the middle of the sulcus, at the position of the vertex. To characterize the position of the motor area in the motor homunculus with respect to the international 10-10 system, 1 cm behind the vertex (Cz) was specifically characterized. Another more precise way to characterize this representation is to use TMS to elicit the MEPs of the desired area and characterize the position in the motor cortex of the desired hemisphere. Because of the characteristics of the lower-limb representation in the motor homunculus, it may be difficult to isolate certain areas without activating others [79].

The predisposition of a specific laterality in tDCS work is mainly due to the special conditions of stroke patients as there is a hemiparesis due to damage of one hemisphere affecting the contralateral musculature. In this sense, there is a stimulation setup that has been become more popular in stroke studies [28,80], which is based on increasing the activity of the affected hemisphere and decreasing the contralateral hemisphere, since excessive plasticity in this hemisphere could be counterproductive [81] and could cause interhemispheric inhibition [82].

Depending on the type of electrodes, different areas can be stimulated at the same time with less or more precision; this will be discussed in the next section. The distance between the electrodes is a variable that has an impact on the intensity field. It has been shown that the greater the distance, the greater the intensity of the field [83], but conversely, the less the focus on the target region [84].

In the literature, the predominant electrode setup is the so-called “conventional setup”, consisting of the placement of sponge electrodes with a typical surface area of 35 cm${}^{2}$, one positioned in the vertex (typically left or right) or motor area of interest and the other positioned in the frontal area typically contralateral to the affected area. Within this set-up, there is some variability in the exact position of the anode-cathode. However, there are other set-ups. A description of some of the set-ups found in the literature can be seen as follows.

**Figure 2:**
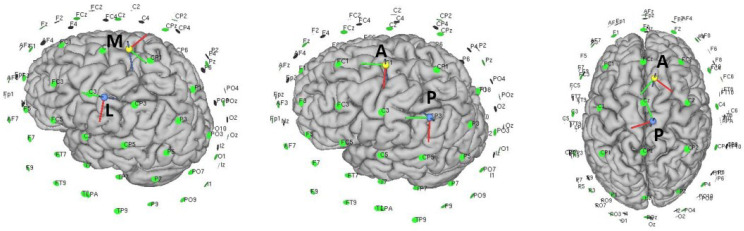
Different types of electrode positionings with respect to the upper-limb area in the first two images and the lower-limb area in the last image.

- **Stroke**
  As mentioned above, stimulation in stroke patients is highly dependent on the side affected by the stroke. In these types of patients, the most common configurations for the treatment of motor function are ipsilesional anodic tDCS, cathodic tDCS of contralesional, and bilateral configurations. Among the 20 papers found, 18 used electro-sponges setup:
  Two papers showed a configuration with the anode centered at the vertex and with the cathode in a supraorbital position.
  Eleven papers presented a conventional ipsilateral configuration and the cathode in supraorbital positions. Four of these papers used TMS hotspot to place the active electrode in the desired position. Among these, two of them made a comparison between setups:
  – In [33], a comparison between ipsilateral anodal with the reference at supraorbital and contralesional cathodal stimulation with the reference at supraorbital was performed. tDCS condition did not perform better than sham. In addition, cathodal stimulation obtained negative results.
  – The paper [44] provided a comparison between ipsilesional anodal, contralesional cathodal and bilateral. Improvements were obtained between the tDCS and sham conditions but not between the tDCS conditions.
  Four of the studies [35,36,37,45] exclusively had a bilateral setup with ipsilateral anode on leg representation in the cortex and cathode in the contralesional, and all of them showed positive results.
  Of the non-bipolar studies, a monopolar study without return electrode in the cephalic area was presented in [46], which compares three stimulation groups: anodal ipsilesional, contralesional cathodal, and bilateral. No differences were reported for the groups between the different setups.
  Only two papers presented an HD electrode setup:
  In [48], contralesional anode electrode setup was proposed with anode at Cz and cathode on the contralesional side (FP2/FP1) and contralesional anode (cerebellum 2 cm right/left). This configuration was based on one previous research tested in healthy subjects [70] with success.
  In [47], two rings were compared, one with the anode in ipsilesional position; C2 (hotspot); and the cathodes in F2, F4, Pz and P4, and the other with cathode in contralesional, for example, C1 (hotspot) and the cathodes in Fz, F3, Pz and P3. Non-significant results were reported.
  Most of the papers found in the literature presented a conventional electrode setup. On the other hand, from the works that inhibit totally the contralesional area [33,44] or partially [47,48], only in [44] positive results were obtained with respect to the sham group. As already discussed, it has been shown that in stroke work, exciting the affected limb and inhibiting the opposite one is one of the best strategies to promote positive plasticity. Positive results have been obtained in the four bilateral bipolar studies [35,36,37,45]. Therefore, bilateral configurations may be more suitable in these cases, as explained above [17,36,85].
- **Spinal cord Injury**
  Two papers reported a conventional setup: one of them [49] with anode on Cz and cathode at the front and the other one [50] with the cathode moved at the non-dominant supraorbital area. At Raithatha et al., 2016 the anode position was chosen at the hotspot position; this one obtained positive results, while the other one obtained non-significant results.
- **Healthy**
  Many of the papers presented in healthy subjects try to test patterns that were then tested in stroke patients. Therefore, many of them tried similar configurations to the conventional setup in a specific laterality. In this case, the non-dominant hemisphere were chosen as the ipsilateral side to avoid ceiling effects. Additionally, depending on the parameters of the task, such as the difficulty, there could be a difference between hemispheres due to the dominance of one in the control [86]. In this section, those works that presented improvements and analysis on studies for healthy subjects that can improve in some way with conventional configurations were chosen for their analysis.
  First, the weaknesses of the traditional assembly were exposed. In [55], the cerebral excitability of the conventional setup with an anode centered on left Cz and a right contralateral supraorbital cathode and then the opposite side setup were analyzed. MEPs activation was evaluated in the muscle group of the leg contralateral to the stimulated side; only 60% of the muscle groups evaluated measured an increase in MEPS in the ipsilateral side. Moreover, down-regulation was observed in 18% of ipsilateral tested muscles, which could indicate hyperpolarization of some areas. In addition, activation of the muscles contralateral to the stimulated area was observed in 40% of the cases, indicating that the current flow in the stimulated area could be deviated to the opposite hemisphere and depolarise it. This may indicate that a long sponge electrode may stimulate the area inaccurately and with unwanted effects on the active and nearby stimulation area.
  The normal component of the flow plays a relevant role in excitability changes. The active electrode is placed on the surface, and the flow is presumed to be perpendicular. However, the cerebral grooves mean that the current does not penetrate uniformly and is difficult to control [87,88]. The tangential component may also be playing an important role in the excitation and the EF distribution [89]. In [90], an electrode montage for the M1 was proposed based on finite element modelling, with the anode 5 cm posterior and the cathode 5 cm anterior to the motor cortex, predicting higher mean EF than standard configurations.
  This setup was tested in areas of the upper-limb cortex representation [73]. In this work, it was proposed that the relative direction with respect to the cortical columns can be determinant in the response of the tDCS. Thus, changing the typical anode over C3/C4 by an anterior-posterior (AP) and medio-lateral (ML) (see Figure 2) anode cathode montage over these areas and trying to create a stimulation flow that goes parallel or perpendicular along the cortical surface would orthogonally affect the pyramidal neurons columns. However, in this work, a comparison with a traditional setup was not performed to test whether there was increased CME activation. The setup AP obtained higher elicitation of MEPs than the ML in this area. In addition, the cathode-anode relative position was also studied for the best setup AP. The best values were obtained for the anode-cathode in position PA.
  Returning to the lower-limb, in [51] an anode configuration posterior to the Tibialis Anterioris (TA) point and a cathode anterior to this point was proposed. In an attempt to corroborate the assumptions of the previous mentioned works, the CME was compared with respect to the traditional setup, and no positive results were reported. To understand why the results were not positive, note that the relative position with respect to the stimulation area of this assembly for the lower-limb is very different than for the upper-limb. An inversion of the anode-cathode position could modify the results in a similar way to the previous study.
  Other studies have tried to propose modifications to the traditional assembly, related to the possible problems that this could have inhibiting unwanted areas. Two solutions have been proposed in [52]: reducing the size of the anode to be more focal (less current above the contralateral hemisphere) and changing the position of the cathode, which would allow one to modify the angle at which the current flow enters the cortical surface.
  Likewise, the proposal of Foester et al. [52] is based on a previous work [91] in which simulations validated several conclusions:
  – The direction of the EF is relevant for targeting the fiber tract.
  – The returning electrode is more critical for small electrodes at motor cortex [83].
  – The returning cathodal electrode placed at the ipsilateral side of the stimulating anodal electrode targets the pyramidal tract fibers better than the traditional montage.
  – The position and size of the returning electrode affects the distribution of the EF throughout the cortex and may change the distribution of the EF in the cortex directly below the active electrode [78].
  Returning to [52], the conventional 35 cm${}^{2}$ long anode assembly was compared using TMS and MEPs versus a smaller anode assembly. The long anode setup was found to be less focal and less effective at generating amplitude MEPs in the desired area than the small anode setup. When the cathode was changed to a lateral position, in the same hemisphere as the anode (in this case T7), more focality and more MEP amplitude were obtained. In addition, the specificity (see Equation (1)) was higher for the T7 cathodal montage than for the conventional setup.
  (1) $$Specificity=\frac{MEP_{targeted}-MEP_{contralateral}}{MEP_{targeted}+MEP_{contralateral}}$$
  The optimization of the relative position of the active-reference electrode was optimized in [52]. The aim was to focus and avoid contralateral activation. Positioning the return in the same hemisphere can be critical, especially for small electrodes. This is very interesting, mainly in stroke setups, where the conventional design may not be performing optimally [55].
  Moving to another cephalic area, three studies studied the role of the cerebellum, and there were two works with monopolar stimulation 3 cm to the inion right or inion left over the hemisphere [13,62,86].
  Switching to HD configurations, six works designed to target the M1-lower and cerebellum were found:
  In [67], the electrode setup targeted the left hemisphere. The anode was located in the sensorimotor area between FC1 and Cz, and the cathode was positioned 3 cm to the left of the inion. The idea behind targeting the cerebellar motor area pathway in such a way was that the motor area was excited and the cerebellum was inhibited in order to produce a benefit in MI. In [69], a similar setup, following this principle too, was proven with other MI protocols. This setup was tested in different polarity configurations [70]. The performance of the polarity in the different configurations will be analysed in Section 3.7.
  In [66], a hybrid configuration was reported that seeks to stimulate two areas simultaneously, the dorsolateral prefrontal cortex (DLPFC) and the motor area related to the legs. To achieve this, an HD stimulation setup was proposed with two anodes: the one directed to stimulate DLPFC was placed in F3, and the other one directed to stimulate M1 was placed in Cz. The returns were chosen close to these areas: FC5, FC1, AF4 and CP1.
  Only two papers reported the typical HD 4 × 1 ring configurations, both with the anode centered at Cz. In [68], a X configuration was used with the cathodes closed together. In [65], a + configuration was used with the cathodes 3.5 cm away from the center.

### 3.6. Electrode Size: HD Montage vs. Conventional Sponge

As discussed in the previous section, the conventional electrode setup has several limitations. Size is one of them, as focality is more difficult to obtain with large electrodes as the electrical field propagates from a larger surface. Another limitation is that the polarity can only flow in one direction with one return electrode. There is another method of stimulation that has gained popularity and overcomes some of these problems. High-definition tDCS (HD-tDCS) is a technically enhanced version of tDCS, which is believed to be more focal [92], better sustained, and longer-lasting in terms of its effects. HD-tDCS is proposed to primarily offer better targeted focal cortical stimulation/inhibition and better cortical penetration at the desired area [15]. In addition, with these assemblies it is possible to configure the areas through which the flow could pass, stimulating two non-proximal areas at the same time [66].

Conventionally bipolar 1 × 1 tDCS setups assume active stimulation on one electrode, while the effects of both (anodal and cathodal) across the brain are present. The distribution of anodal and cathodal stimulation can be modulated depending on the return electrodes included. HD-tDCS stimulation can modulate the stimulation polarity in favor of the center electrode [76]. In this study, the influence of cathodal and anodal current intensity distribution on the stimulated area was tested. The symmetry between cathodal and anodal stimulation was derived from the perpendicular component of the EF, and lower level of asymmetry was paired with higher intensity of anodal stimulation. It was determined that anodal and cathodal stimulation were more symmetrical in 1 × 1 than in 4 × 1. The most significant difference occurred from 1 to 2 return electrodes. Subsequently, the radius was modified in the case of the 4 × 1 ring. Larger radius had a higher level of symmetry than smaller radius but never reached the level of the 4 × 1 configuration. However, this work did not take into account the complex dynamics of motor circuits. In [36], a bilateral configuration with anode was applied over the ipsilateral area. In a single session, positive results were obtained on strength assessment scales for both legs. This improvement is opposite to the fact that the cathode was placed over the contralateral hemisphere. This paper proposed that the bilateral setup may be producing low cathodal inhibition due to the characteristics of the tDCS stimulation setup.

An important topic for which HD stimulation is increasing in popularity is focality. Focality is usually defined as the narrowing of the spatial distribution of the cortical EF in relation to the peak. The focality on the stimulation area is very low in monopolar configurations, higher in bipolar configurations and can increase depending on the HD configuration chosen (number of return electrodes).

Regarding focality and EF peak relation, as opposed to the conventional setup in the 4 × 1 configuration, the maximun EF field appears under the stimulation area [92]. In the conventional configuration, it would be midway between the active and return electrodes larger than 3 cm rather than below the stimulation area [93]. Focality cannot be discussed without taking into account the intersubject variability that this focality may produce. In a recent study with healthy subjects comparing EF magnitude [94], focality and EF variability among subjects using MRI computational models on the hand-M1 area, 13 electrode assemblies were studied. The parameters mentioned were compared for the TMS setup, the conventional sponge setup with different sizes, the conventional sponge setups with circular anode, the HD ring setup and the HD ML and AP setups. The conclusions obtained were: the focality and EF magnitude increased as the size of the stimulating electrode decreased. In contrast, the variability of the EF was highest with small electrodes and decreased with bigger sizes at the cost of increasing variability. The TMS obtained values of focality and EF magnitude were similar to the ones of 4 × 1 HD and greater than the best values obtained for the conventional setup. However, the TMS was the second assembly with the lowest variability and the ring one was the highest. The ring setups were the ones that had the most variability, and the setup with more anode-cathode distance obtained less variability and greater EF magnitude than the setup with closer cathodes.

The highest focality values were for the ring configurations; between the rings, the narrowest ring achived the best focality values. However, the variability was very high. The best variability values with a good relationship of the EF magnitude parameter were in the ML and AP assemblies. The ML setup was the one that obtained the best relation of variability and EF magnitude, with better focality values than the AP.

Nevertheless, as individual anatomy also affects tDCS, EFs increasing the focality of stimulation could result in greater uncertainty in group-level results due to EFs being more affected by local individual anatomy [94,95].

Although these results may be of interest to our research, they must be interpreted since, as has been mentioned above, the direction in which the flow crosses the walls of the motor homunculus is different for the areas of the hand in which the pyramidal neurons are perpendicular to the scalp, while those in the leg area are horizontal with respect to the scalp. Furthermore, high reliability and low variability do not necessarily indicate the clinical efficacy of tDCS [54].

Another important factor is that current density decreases in deep cortical areas in a faster way for small electrodes, for which higher currents may be needed to reach deeper areas in HD configurations than in traditional assemblies [83]. In [92], it was estimated that the intensity needed to have the same magnitude effects of the EF as in a conventional setup should be twice the one obtained in a 4 × 1 ring configuration.

Regarding the time in which the tDCS can have an effect on the modulation of plasticity, only one study in the literature compared the effects of HD versus conventional tDCS on the position of the hand in the cortex [15]. According to this study, conventional tDCS has an immediate effect, while HD-tDCS has been reported to start 30 min after. In addition, the descent time would also be variable, while tDCS conventional could decay after 120 min, and the HD-tDCS would have a decay after 6 h.

The different mechanisms that HD-tDCS can produce were compared to conventional tDCS and the latest advances. The works found in the literature that explored different sizes or that tested some HD models focusing on lower-limb setups will be discussed in detail:

- **Stroke**
  In [46], monopolar sponge electrodes with an area of 1.75 cm${}^{2}$ were used. The study did not obtain significant results between groups but individually. It was found that gait parameters improved for all configurations as soon as the session ended. These results may be related to the analysis carried out in [94], and a reduced electrode size increases the focality and increases the variability among subjects.
  In [47], a ring-shaped setup was evaluated, which aimed to partially inhibit the contralesional area and excite the ipsilesional area. This work did not obtain positive results in the functional scales measured compared to the sham group. This work tried to replicate some of the positive results obtained with bilateral assemblies in a single session of anodic tDCS, in other works [35,36,37,45]. The authors attributed the negative results to the fact that the montage was not forcefully inhibiting the contralesional hemisphere. The 4 × 1 focused setup with HD tDCS failed to replicate the results in bipolar sponge setups.
  The study of [48] with 6 subjects showed negative results in an HD configuration whose objective was to excite Cz and cerebellum and inhibit the contralesional side.
- **Healthy**
  The abovementioned study [52] with sponge electrodes corroborated the hypothesis raised in [94], on focality, although it did not report a clear comparison of the variability among subjects for each type of setup. Small electrodes on the desired area obtained more MEPs in the target area and fewer in the contralateral area. Therefore, this study supported the hypothesis that smaller electrode configurations can focus the effects of anodal stimulation on the target area and reduce stimulation on the contralateral side. Configurations of this type, such as HD setups, may improve the results obtained so far with conventional setups. Among these configurations, one of the most popular is the 4 × 1 ring configuration.
  To our knowledge, only two works have been reported for the lower limb in healthy subjects, and only one has commented on stroke patients [47], with the results being not very promising. Regarding the other two, the research in [68] did not report relevant results, and in [65] only one parameter seemed to improve. However, these two studies used opposite current intensities, the first very low and the second very high. Therefore, the possible variations in focality may be offering highly variable results [94].
  There are three works that use the HD-tDCS property to stimulate several pathways simultaneously that have reported significant and promising results. The Cz-cerebellum pathway [69] obtained inferior results to those of the Cz, cerebellum-FC2 pathway [70]. Another work, which has also shown promising results, can be seen in [66]. The research stimulated mixed areas: the cognitive one centered in the dorsolateral prefrontal cortex (DLPFC) and the motor one in M1.

### 3.7. Anodal vs. Cathodal Stimulation and Time Dependent

The polarization, direction parameters and flow may have different properties regarding neuronal activity, depending on the active and return electrodes. Monopolar stimulation provides a roughly radial current diffusion that covers an approximately spherical space around the stimulating electrode with a relatively high volume of tissue activated during stimulation. In contrast, bipolar stimulation creates a narrower and more focused current field. However, it produces a certain polarization on one part and the opposite polarization on cephalic positions. The number of return electrodes can modulate the distribution of polarization over the stimulated area.

Although the anode is often used to increase neural excitability and the cathode to inhibit it, under certain conditions this behavior can change. In [96], a 2 mA anodal stimulation (0.06 mA/cm${}^{2}$) during more than 26–30 min resulted in inhibition, which may be due to a neuronal counter-regulation of calcium mechanism, which prevents over-excitation. Otherwise, the application of a 2 mA (0.06 mA/cm${}^{2}$) cathodal intensity stimulation for 20 min results in cortical excitability enhancement instead of inhibition [97]. There is a biomedical explanation for this effect, as the high intensity of the cathode can increase calcium levels and therefore induce LTP plasticity, which is in line with Monte-Silva research [96]. Another is be that, due to the increased flow, structures with a different neural orientation can be reached. Understanding stimulation as a modulator of noise may help to explain this phenomenon from another point of view as these effects could be the result of an inhibition of noise in the secondary circuit [98].

- **Stroke**
  The role of anodal and cathodal stimulation is very well defined in this type of study, as it has been previously discussed. However, this modulation can be affected depending on the affected networks. In this review, the different inclusion criteria have not been taken into account depending on the brain area affected by the stroke. As mentioned in [99], depending on the severity of the stroke, if the corticospinal tracts are affected, plasticity may not be effective and therefore M1 ipsilesional anode stimulation may be ineffective. In addition, contralesional cathodic stimulation could be detrimental due to the need for the intact hemisphere to take up certain motor functions from the injured hemisphere.
  The following works showed problems that could be partly due to this. In [33], an ipsianodal stimulation was compared with contralesional cathodal stimulation, hoping that contralesional cathodal stimulation would favor learning. However, this study reported unfavorable results in contralesional cathodal stimulation and did not obtain improvement results in tDCS versus sham. Due to the characteristics of the setup presented in [48], inhibiting the contralesional side could explain the variability reported for some of the subjects. However, in [99] it was also stated that anodal stimulation on the contralesional side could be beneficial for some of these cases. This partially contradicts what has been explained so far about the importance of not stimulating the contralesional side, and it has been seen that it has non-positive effects. In [29], it was reported that stimulation with an ipsilesional M1 leg cathode improved the task of tracking the ankle, while M1 stimulation of the contralesional leg produced negative effects on learning. However, partially supporting this hypothesis, results obtained with the ring configuration of [47], with the cathode centered on the ipsilateral zone and the anodes centered in the middle, were modest regarding the measurement of ground reaction forces and showed negative results on other indices.
- **Healthy**
  To our knowledge, one of the first studies that explored the effects of tDCS on the leg area of the motor cortex was the work carried out by [53]. In this work, the excitation of the motor cortex was measured through the measurement of monopolar anodal, cathodal and sham stimulation MEPs during contraction and rest tasks. Anodal stimulation seemed to increase excitability in both states, while cathodal stimulation did not seem to inhibit with the same force. Only a small difference was noticed with respect to sham in the rest state. Monopolar anodal stimulation on Cz showed MEPs elicitation differences in comparison with cathodal and sham. This work showed the possibility of exciting the cortex. It is more difficult to elicit an inhibitory response than an excitatory one in the muscle groups related to the lower limb.
  In [55], an evaluation of MEPS was made and the efficacy of eliciting MEPS for the lower-limb with the upper-limb was compared. The percentage of activated muscles in this study, 60%, was lower than in those of the upper-limb. Among the muscle groups that were not activated, the most likely explanation was that the RT failed to activate all the fibers. Using conventional tDCS to cause anodal stimulation in the motor leg homunculus is a more complicated issue than in the upper limb. Until now, it has been discussed how the HD and the relative position between electrodes could produce more focal results in this area. However, it was also hypothesized that for those groups, the hyperpolarization of the neurons in synapses with the neurons of the pyramidal tract in deeper layers gave rise to the down regulation of the excitability of the motor system in this sense, and controlling this effect could be more complicated. However, this phenomenon could be due to an angle of the field that crossed the wall of the homunculus in undesirable directions, creating a hyperpolarization in some fibers.
  Moving to the cerebellum area, research findings have found that tDCS over the cerebellum produces cortical excitability changes in a polarity-specific manner [100]. In general, anodal stimulation is believed to enhance motor and cognitive functions, whereas cathodal stimulation typically inhibits them. However, the parameters under which this polarization is achieved are not completely defined. Not all works have been able to replicate these results [101]. Works more focused on cognitive function used a montage from the back of the head found no polarity-specific effects. However, those more focused on motor function that used a montage from one side of the back of the head found polarity specific changes [102]. The latter are described below.
  The role of the cerebellum in the lower limb role has been studied in [86]. A monopolar anodal, cathodal or sham tDCS configuration was tested on the representation of the leg dominant cerebellum during the performance of a motor task on a split-belt treadmill. In [63], the connectivity between M1 and cerebellum was evaluated using the cerebrall-brain inhibition (CBI) indicator. It was reported that modulation in brain excitability could affect learning adaptation. CBI was reduced after learning by LTD changes. Although it would be expected that cathodal stimulation would facilitate LTD by improving learning, it is not always like that. In the experimentation carried out in [86], positive results were obtained for anodal/cathodal stimulation versus sham stimulation in the adaptation ratio of the spatial elements of the walk. The ineffectiveness of cathodic stimulation on the cerebellum was also corroborated in [62] during a gait adaptation task.
  These results have been corroborated in a series of studies related to EEG and HD-tDCS in which a BCI was proposed to differentiate between mental states. Cathodal stimulation did not obtain positive results, while anodal tDCS did. In [67], the effect of different intensities was evaluated in several individual sessions. Cathodal stimulation on the cerebellum did not obtain significant results. In [69], a configuration similar to the one mentioned was tested for 5 days comparing the sham and tDCS group. No differences were obtained in the improvement of the task for both groups. The tDCS group obtained similar results to the sham group but with an anticipation in days. In contrast, the anode setup on cerebellum [70] obtained positive results with large differences in accuracy for the tDCS group. In this work, two BCIs were tested: one with visual feedback and another with the motor feedback provided by the exoskeleton. Furthermore, the BCI-motor feedback experiment obtained higher results for tDCS and sham groups than the BCI-visual feedback one. This work, in addition to having the objective of inhibiting on the contralesional side to excite the ipsilesional side in its application in patients [48], by inhibiting in FC2 or FC1 areas it has been shown [103] that an excitation effect can be achieved in Cz and somatosensory area. These results also go in the direction of what was demonstrated and predicted in [102,104], where it was hypothesized that anodal stimulation can be effective for strategies focused on adaptive learning.
  These strategies have shown efficacy in learning MI during days of training in a task that required motor learning (visual feedback) when stimulation was performed anodally versus cathodally. However, in [13], it was shown that cerebellum anodal stimulation inhibited upper-limb motor imagination compared to the sham group after a single stimulation session. It should also be taken into account that in Ugarte et al., at the same time that a-tDCS was applied on the cerebellum, it was also applied on the area of M1 (Cz); this could have balanced the expected depolarization in this area.

### 3.8. Current Strength

The tDCS produces a subthreshold stimulation when a weak current results in a biphasic polarization [105]. A correct selection of intensity is important to reach the expected cortical areas and to achieve an adequate polarization.

Therefore, depending on the other factors mentioned so far, such as the number of return electrodes, size of electrodes and distance between them, the current penetration intensity and the magnitude of the EF may be altered significantly and must be adjusted to achieve the expected results.

However, an increased current density is not necessarily paired with increased brain modulation [77]. The high conductivity of the scalp and the cerebrospinal fluid, relative to those of the skull and the brain, makes the current to flow tangentially through these tissues, limiting the flow current in the aimed brain areas [83].

Current density is the measure used to establish the range of suitability in which tDCS is effective and safe for subjects. Brain injury is predicted to appear at brain densities of 0.63–1.3 mA/cm${}^{2}$ [106]. In addition to avoiding setting current densities that could be close to neural damage, it is also important to consider the currents that may be generated on the scalp.

The 4 × 1 ring configuration produces a higher surface current that does not lead to a higher peak-induced cortical EF magnitude as it reflects and flows across the scalp without crossing into the brain [92]. Depending on the distance between the electrodes, the percentage of shunted current varies. In MRI studies [83], it has been simulated that 35% of the injected current may reach the brain if the electrode distance is around 8 cm and more than 60% when the distance is over 20 cm. The current shunted through the scalp and the cerebrospinal fluid is unlikely to be harmful. However, if too much current is shunted through the scalp, the stimulation may become less well tolerated as a high current in this area could produce an unpleasant sensation.

In the literature, most papers report current densities around 0.05–0.08 mA/cm${}^{2}$. As discussed in HD stimulation, this current is usually increased. Among the assemblies using sponge electrodes, few have been reported above the 0.08 mA/cm${}^{2}$ limit. Slightly above this value: 0.13 mA/cm${}^{2}$ [30,56]; above: 0.28 mA/cm${}^{2}$ [31]; and well above 1.75 mA/cm${}^{2}$ [46]. Among the works using HD electrodes and configurations, the current densities are higher on average than that of conventional configurations. In the work of [70], anodal stimulation corresponding to 0.16 mA/cm${}^{2}$ was used. For ring configurations, it is estimated that the current density should be higher. Except in the work of [67], other configurations apply higher current, such as 0.44 mA/cm${}^{2}$ [47] or 2.54 mA/cm${}^{2}$ [65]. In the work of [66], two anodes were used, each one with a density of 0.47 mA/cm${}^{2}$, which is equivalent to a flux density of 0.94 mA/cm${}^{2}$.

Intensity is a factor that has not received much attention in terms of specific variations. There are simulation models that allow one to determine the optimum intensity depending on the configuration. However, none of the works in this review use it.

Another relevant factor is the duration of the stimulation. Time is an important factor since it can have repercussions on the level of polarization. Among the works found, the most used time intervals were 20, 15 and 10 min, reporting a 7 min excitation only one work. Eighteen studies applied a 20 min stimulation, in 13 studies for 15 min and in 11 studies for 10 min. Knowing the number of electrodes, the intensity of each one and the time, it is possible to calculate the value of the charge received by the individual during the whole session, called dosage.

### 3.9. Offline Vs. Online

The mechanism under the explanation that anodal tDCS increases excitability and cathodal stimulation decreases excitability is rooted in the evidence that tDCS modulates membrane potential and somas. However, these theories do not go into special detail on what could be the differences between the effects of offline and online stimulation during motor learning. There are theories that partly explain what anodal and cathodal tDCS could produce in the absence or presence of cerebral endogenous activation. The BCM theory states that when a presynaptic neuron fires, the postsynaptic neurons will suffer LTP if they are in a state of high activity or, conversely, LTD if they are in a state of lower activity. However, this theory is not fully corroborated.

Other proposals show some disagreement with these bases; in [12] it was mentioned that the polarization gradient on the membrane could play an important role in the regulation of endogenous plasticity. Endogenous NMDAR could be an elemental key for anodal and cathodal tDCS to exert a modulating role on plasticity. The activation at the apical or basal level of the dendrites during anodal or cathodal stimulation can also produce variable effects. In this way, the variability observed in many of the works could be partially justified. In this work, it is proposed that tDCS would be a regulator of plasticity rather than a generator.

Although the biological mechanisms of tDCS during learning are not completely defined, online vs. offline relevance of stimulation for therapy is discussed in some of the literature works. Some of them applied the tDCS in a semi-online way, before the main task but paired with a similar motor task.

- **Stroke**
  Only one study compares or allows for a comparison of the offline and online states in stroke patients. In [42], the effects of a traditional tDCS setup were tested in a treadmill training for four control conditions: control, AMT, tDCS, and tDCS+ AMT. There were no significant differences in the kinematics parameters related to the behavioral task. Changes in CME occurred at the paretic side, for the tDCS+ AMT group.
- **Healthy**
  The effects on cortical excitability, which anodal stimulation in M1 can produce depending on whether or not a task is being performed, are usually supported in the literature by BCM theory.
  In [53], cathodal stimulation showed different behavior depending on whether the stimulation was done offline or online, while anodal stimulation obtained higher CME values in the offline trial than in the online trial. Although the dominance of the online group was observed once the task was finished, later an equilibrium of the online and offline groups was observed. In [56], cortical excitability and task accuracy were tested for a single session during an ankle motor tracking task for three conditions: a-tDCS before the task, a-tDCS during the task, and sham. The evaluations were carried out during practice in four points and at the end in three different conditions. During practice, the online group obtained significantly higher results than the rest of the groups. The offline group reduced activity during the task, with equal or worse results than the sham group. Between 20 and 25 min later, this trend continued 24 h later: the offline and online groups obtained the same percentages, both being higher than the sham group. The results of the CME were not significant. In [54], an attempt was made to perform a more precise measurement of excitability presented in the previous study. Differences were obtained between the mean of the CME of the online group with respect to the offline one. However, [53] reported the opposite conclusion. The task performed in this work is not specified, so these differences cannot be argued.
  The effects of stimulation could also be task dependent. Saruco et al. demonstrated in two studies the differences of performing stimulation before or during a balance MI task [57,60], although in all groups there was some improvement. The most optimal combination for a greater before–after test difference was observed in the group that applied tDCS during the MI task. In both studies, it was concluded that these results could support the BCM theory.
  At [66], three tDCS states were performed prior to the task. The tDCS+seated versus tDCS+walking groups were compared. In the tDCS+walking and sham+walking groups, differential results were obtained for both dual-task cost (see Equation (2)) and gait speed dual-task. For the tDCS+seated condition, the results did not improve but worsened compared to baseline. The results were significantly worse than the tDCS+walking group. An important factor, with respect to other works, could be the type of task that is executed during the stimulation, such as the Madhavan’s works being an ankle motor task.
  (2) $$Cost=\frac{Speed_{DT}-Speed_{usual}}{Speed_{usual}}\times 100$$
  Although these results partially corroborated certain BCM hypotheses, LTD depression in the offline stimulation group was only observed in [66]. Nevertheless, a 90 min delay could be needed to induce shift adaptation [107,108].

In the community, there is a belief that online a-tDCS is favorable. However, depending on the task, very variable results were shown; some of the cases of tasks whose works in the bibliography are more easily comparable are discussed below. For example, for AMT it seems that tDCS modulate excitability and task performance favorably when applied online. Several of the works by Madhavan et al. corroborate this [54,56]. In addition, in two studies [42,66] with different protocols, it is discussed how using tDCS+AMT to measure performance in a Treadmill Training (TT) task can produce positive effects. In [42], CME changes were observed, but only in [66] were changes in task behavior were observed. Additionally, in the work of Saruco et al. [57,60], it was observed that MI accompanied by a-tDCS was better than offline stimulation. Regarding a robotics task, the online application does not work well. Online works [41,43,50] have obtained negative results, while those who applied offline have obtained positive results [38,39,49,70]. A more comprehensive case comparison was performed in [109], where the temporary combination of tDCS plus robotic therapy for patients with chronic stroke in the upper limb was studied. Giacobbe et al. Argued that tDCS applied before robotic assist training led to improvement, but tDCS during or after did not improve the results. This topic will be covered in more detail later.

The effects of tDCS on the combination of offline or online events can be highly variable and task dependant. No clear patterns were observed in the literature. In the following sections, a more detailed analysis of the results will be applied according to the type of measurement and task.

### 3.10. Time-Dependant Effects in Single and Multiple Sessions

The tDCS can be designed as a single trial in which the results are observed during stimulation or in the short term immediately after completion. The effects of tDCS in a single session have an effect with a range of approximately one hour for sponge stimulation and 6 h for HD stimulation [15]. However, if repeated cumulatively, an increase in long-term plasticity and learning and a long-term increase in these benefits are expected. To measure these cumulative effects, measures should be reported after each session or at the end of the test, weeks to months later. In those trials that measure the ongoing effects of tDCS, they should be measured at the progression of each session.

The ultimate goal of tDCS is to design effective rehabilitation therapies, which in the clinical setting can improve on conventional rehabilitation therapies, accelerating this process and allowing the patient to recover sooner and better. tDCS therapies accumulated over days seem to be better adapted to this principle; however, understanding the short term effects is also an important task to better understand the effects, and this can help to identify improvements to be made in order to generate more solid long term therapies. Among the works that carried out sessions for several days, in the three Tables there is a distinction between those that carried out the sessions consecutively c (Monday to Friday) or intermittently s.

- **Stroke**
  First, tDCs works for single session are described, starting with those who reported CME measurements and later for those who report behavioral averages. CME excitability has been tested paired with an AMT task [29], and anodal stimulation with the conventional setup increased excitability during the task and decreased excitability on the contralateral side. High-Intensity Interval Training (HIITT) practice has been reported to decrease excitability in some patients, and the combination of tDCS+AMT and subsequently HIITT has been reported to increase excitability in a single session [30]. With respect to those papers measuring physical behavioral outcomes for a TT task with monopolar tDCS individuals, subject results were obtained but not significant results [46].
  The following are the works that fail to replicate tDCS in a single session. In [33], no significant results were obtained in one session of tDCS in the balance and gait performance parameters, as in other studies [46,47]. However many others works have shown significance [32,34,35,36,45]. In [32] the evaluation was performed 1 h later. In this sense, there is quite a mix between post-evaluation measurement times, which could be influential and could be variable depending on the offline or online model of stimulation chosen. For example, in the work of Tanaka et al., online tDCS was applied during force knee, and significant effects were observed during the performance of the task but only up to 30 min after this.
  Regarding the works that study the long-term effects, another stimulation study tried to prove the effects reported in [29,30] but in walking parameters. In [42], a HIITT training was performed for one week; in none of the tDCS groups were differences shown compared to the control groups, and no significant improvement was shown in walking outcomes either during the curve of the days, once finished, or three months later. However, as reported in previous studies, instantaneous changes were observed, as well as cumulative changes in the CME parameters.
  Like in [42], many other papers reported no improvement in gait parameters [40,41,43,48]. As already discussed in this review, there are a multitude of factors that may differentiate these works from those that obtained positive results; however, the feasibility of tDCS under certain conditions is a topic that requires further investigation.
  Among the studies that have shown positive effects, four of them showed long-term effects from 1 month to 3 months after stimulation. In [31], significant differences were shown right after the end of the sessions between the sham and tDCS groups on the Functional Ambulatory Category (FAC) scale. The differences between groups increased on the FAC, and especially 6 Min Walk Test (MWT) scales, 4 weeks after treatment. In [39], there was significant differences in the FAC, Time Up and Go(TUG) and Stroke Impact Scale (SIS)-16 scales; 1 month later all scales maintained similar values. In [38], there were significant differences at the end of the sessions between the sham and tDCS groups in the FAC scale. The differences between groups increased in the FAC scales, and especially 6MWT, 4 weeks after treatment. In [44], for balance measures related to the risk of occurrence falls, significant improvements were obtained at the end of treatment and up to 3 months later, all of them in offline regimen.
  However, in many of the studies reporting positive changes, many parameters did not show positive results.
- **Spinal cord Injury**
  The authors of [49] conducted a measurement of the scales just after finishing the therapy, and 1 month after the first intervention significant results were obtained in the strength scale.
- **Healthy**
  Of the works found on healthy people, out of 22 works, 7 were applied during consecutive days. In healthy subjects, there are many more papers that are applied in a single individual session. Demonstrating cumulative effects in healthy subjects can be complicated due to ceiling effects.
  Single session CME measure was evaluated in [53,54,56]. None of these works concluded that CME had significant variations between online and offline tDCS application. Post-task changes were observed in anodal CME with respect to sham, and in cathodal, although these more discrete and dependent on the type of stimulation.
  For a single session with older adults [58], balance did not obtain significant values on the Time in Balance (TiB scale). In contrast, it has been proven that a single session of anodal stimulation [57,60], together with a motor imagination task in online mode (also offline although to a lesser extent), can have positive effects on balance.
  In the work of [59], the effects of anodal stimulation on reaction time were demonstrated, while the effects of a high-intensity session focused on the lower-limb [65] did not obtain results on strength.
  Regarding the validation of walking parameters in tDCS combined with TT, of all the papers that tried to test the effects of walking parameters in TT, the paper presented in [66] is of the few one that has positive effects.
  Returning to the cerebellum: the effects of single-session anodal stimulation on cerebellum stimulation decreased motor imagination [13]. However, as discussed in the polarity section, the effects of a single session of cathodal stimulation [86] had no significant effects on a cognitive-motor task, whereas anodal stimulation in a single session did produce positive effects [62].
  Of the works applying tDCS in repeated sessions, only two of them record long-term effects:
  Compared to the single-session work presented in [58], the results obtained in [61] with older adult subjects are promising. The effects were evaluated just after stimulation, 30 min after stimulation and 1 week after stimulation. Differences in the three conditions are estimated for the TUG, time-Modified Figure of Eight Walk Test (MFEW) and steps-MFEW scales only in the last temporal assessment.
  The cumulative effects during a consecutive week of MI + tDCS were evaluated in [69,70]. In the first paper with cathodal stimulation, no significant improvement curve was reported in the tDCS group versus the sham group, while in the second paper significant differences were reported and maintained throughout the week.

Anodal tDCS has been shown to increase cortical excitability during and moments after stimulation, regardless of whether stimulation occurred online or offline. The validation of physical parameters depended on the task: RAG, TT, balance, pedaling, or mobility and physical therapy. Although in the majority of conditions works have been reported that achieve positive effects, opposite results have also been found. This variability may be partly explained by the type of task, the task difficulty and the type of stimulation. In the group of papers related to exoskeletons, there may be a clear pattern to this hypothesis. This will be discussed in depth in the next section.

However, it is possible that no setup will be able to reduce this variability shown by the tDCS. It is known that the spinal central pattern generator is an important mechanism in human gait [110]. The benefits of these techniques in improving gait parameters have been tested with superior results to tDCS stimulation alone.

### 3.11. Robotic Assistance Combined with tDCS

In these section, the works found that use robotic assistance will be mentioned; the only work that used a walking exoskeleton was the work of [70]. The rest of the works used Lokomat.

- **Stroke**
  There were four studies in which a rehabilitation therapy was applied through stimulation and a robot that assisted the patients walking. Of these studies, two applied tDCS in combination with robotic assistance at some time. In [41,43] this was applied during the first 20 and 7 min, respectively, and these two studies did not obtain significant results. In the first, nine subjects were evaluated in the active group versus 23 in the control group, and in the second *n* = 20.
  In contrast, positive results were reported in offline therapies [38,39] with significant improvements in the FAC scale. The first of these papers is the only one in the entire literature on tDCS with robotic assitance that measured CME, with no significant difference in these scales. Both studies had similar conditions, except that in [38] the anode was placed in the ipsilesional hospot (with TMS) and contralateral cathode while in [39] the anode was placed on Cz and cathode at frontal. In the first work, 11 subjects were evaluated in the active group versus 10 in the control group, and in the second *n* = 10.
  Furthermore, as already mentioned, there is some variability between these studies. In [41], the patients evaluated were in the subacute phase, and the position of the electrodes is slightly different from the rest of the studies. On the other hand, in [43] the stimulation was applied only for 7 min, a value below the rest of the studies that therefore could be a critical factor.
- **Spinal cord Injury**
  The two works found consisted of tDCS in combination with robot assistance gait. In [49]; stimulation was applied offline. The tDCS+robot assistance group improved compared to the sham+robot assistance group in muscle motor in the right leg. However, 6Min WT and TUG obtained negative results compared to the control groups. The effects were maintained after 1 month.
  Conversely, in [50], all the scales showed non-significant results between groups, although all groups showed positive results at the end of therapy. The tDCS were applied online, compared to the Raithatha et al. study. This last study was with a sample of subjects superior to the Raithatha et al. study *n* = 24 vs. *n* = 15.
- **Healthy**
  Only one study was found, in which a walking ambulatory exoskeleton was used [70]. The number of subjects was very low *n* = 4. The study reports positive results in the distinction of two tasks, MI and relaxation. These results are also compared with another experiment reported in the same study with *n* = 12 but without the exoskeleton. The exoskeleton experiment resulted in higher results for all groups; in any case, more subjects would be needed to establish a clear analogy. This is the only one of the studies in which a BCI and stimulation therapy were combined.

It is difficult to draw a conclusion between studies due to the high variability and difference in parameters. In the acute or subacute phase, in which the injury is recent, there may be very heterogeneous rehabilitation conditions. This is more significant in stroke patients: evaluations within the subacute phase can be altered by cognitive problems, in addition to the fact that spontaneous recovery can occur at this stage. However, all the studies except one were in the chronic phase. Although all studies had a control group appropriate to the characteristics of the study, the number of balanced subjects per group in all studies was below 25. Among the works that showed positive results at the end of the session, all the works in patients also obtained positive results in the long term. No work reported positive results in CME changes in this type of task.

However, it should be emphasized that, according to the results of [109], no lower-limb study in which tDCS was applied at the same time as partial or total robotic assistance showed positive results. It could be hypothesized that anodal tDCS applied simultaneously with a high-demand task (high excitation) cannot cause LTP, probably because the excitation noise in the circuit is too high. This detailed analysis of the parameters can provide information that may be hidden when all works are analyzed together [99].

### 3.12. EEG Combined with tDCS

The areas of the brain that are activated during a motor task are the supplementary motor areas (SMA), M1, the primary somatosensory cortex (S1) and the premotor area (PM) [111]. During motor imagination, neural pathways related to these tasks are activated in a similar way to during real movement. The cerebellum is also an area that plays an important role in maintaining balance and posture, coordinating voluntary movements, motor learning, and cognitive functions. The cerebellum sends information to M1 through the dentate nucleus. If the Purkinje cells are activated, this can result in inhibition of the dentate nucleus and therefore inhibition of M1. Therefore, the cerebellum plays an inhibition roll in MI [13]. If the stimulation is anodal, activation occurs in the cerebellum and a depression occurs in M1, and in the opposite way for anodal stimulation.

Depending on the area to be activated, motor imagination can be of different types: finger contraction, balance, pedaling and walking. Furthermore, MI is characterized by the decrease of power in the bands high theta (6–7 Hz), alpha (8–12 Hz) and beta (13–35 Hz). This process is known as event-related de-synchronization (ERD) [22], and it is useful to differentiate between MI and relaxation states. These changes in brain activity can be measured by multiple recording methods. In the same way as the tDCS evaluation, all the techniques that measure cortical activity are valid: TMS, EEG and fMRi. In the selection and discard stage, a paper was found that evaluated MI by means of a word test. However, the effects that a task produces on functional improvement after performing the imagination task can also be measured.

Within the techniques of EEG characterization and in the field of BCI, MI is usually classified by differentiating it from other neural activity, which is usually relax. To differentiate this pattern, the EEG is pre-processed with filters that restrict the desired frequency bands. From the signal, patterns are extracted that can be frequency-related, temporal or spatial [112]. Class feature are often modeled with a classifier, commonly support vector machine (SVM) or linear discriminant analysis (LDA). Of the studies found, only three proposed EEG processing.

Based on the combination of tDCS and EEG, the following methodologies can be distinguished: offline, pretend to evaluate the short and long-term after-effects induced by tDCS, and the online with EEG recording performed during tDCS stimulation, to evaluate the ongoing changes occurring during tDCS. These two methodologies produce significantly different effects on EEG recording. In the online methodology, ref [113] increases of low alpha and beta ERD localized in specific areas of the cortex have been observed. Additionally, changes in coherence have been noted in beta and theta bands. However no objective study was found to follow this methodology, and therefore no further detail was provided.

The variation of brain oscillations when applying offline tDCS has also been studied. As investigated in [114], anode stimulation alters ongoing brain activity, specifically in the rhythm of the alpha band. This section will go into more detail about those studies that monitor brain activity:

- **Healthy**
  Two studies with the same procedures [67,68] explored cathodal stimulation in the cerebellum-anodal sensorimotor area and ring stimulation on Cz. Both studies had five users and randomly performed a session with a current density of 0/0.02/0.04/0.06 mA/cm${}^{2}$; between sessions, there was a 2-day gap. The signal was filtered between 5 and 45 Hz, and artifacts were removed using an independent component analysis filter. The extraction of signal features for two states relaxation and MI was carried out using the fisher criterion of the spectra on C3, C4, Cz. The features were classified using an LDA classifier, and the results were the accuracy with which the signal was separated. According to the hypothesis, an increase in accuracy was expected as the current density increased. In both studies, the results were highly variable and not very significant. In [67], cathodal stimulation did not obtain significant results, although cathodal stimulation on the cerebellum would have been expected to significantly improve MI, as can be deduced from [13]. In addition, this work questions whether introducing a learning process in the task (feedback) could improve this paradigm. On the other hand, in [68] it was hypothesized that the low intensity applied for the ring configuration would not have sufficient focality [83].
  Two works with similar procedures also study the changes proposed above. A BCI for the rehabilitation and control of devices is proposed in [69,70]. The BCI is presented in the following way. Users perform a training session in which the brain signal is modeled according to relaxation and MI patterns related to pedaling and walking, respectively. For nine central electrodes, the signal is filtered between 0.5 and 45 and a Laplacian filter and processed by extracting the power signal. A model of the signal is created with a SVM. Subsequently, the BCI is evaluated in real time through a learning task. As already mentioned, the results of [69] do not corroborate the hypothesis that cathodal stimulation may be beneficial for learning an MI-based task. However, the results obtained in [70] support the theory that anodal stimulation applied before motor learning may be optimal to accelerate and enhance BCI learning process.
  In [71], the visual feedback experiment explained in [70] was extended. With the same conditions, partial direct coherence values for the alpha and beta bands were evaluated. The inflow and outflow values for the sham and tDCS groups were analyzed. The inflow value was a measure of the electrodes with the highest ratio of information coming from other electrodes, while the outflow was a measure of the amount of information they provide to the rest of the electrodes. Because of the stimulation characteristics of the study, the left hemisphere should be excited due to the inhibition of the right hemisphere. In the tDCS group, there was a higher inflow interaction than for the sham group.
  In addition, for both sham and tDCS groups a significant output interaction flow toward right or left from SMA, M1 and PM areas (Cz, FC1, FC2, CP1 and CP2) was reported.

## 4. Discussion

This review includes a wide variety of papers, which have very different characteristics that make it difficult to compare their results or formulate any kind of comparative statistics. To facilitate the comparison, the different sections have addressed the most important aspects to be taken into account for the design of a stimulation protocol. Conclusions were drawn taking into account the results obtained from different measurements. In each of the sections, some of the most general aspects were introduced to understand each of the concepts, and then the works found exclusively for the lower-limb were discussed. These works are detailed in Table 1, Table 2 and Table 3. The most relevant aspects to be taken into account in the design of stimulation protocols are discussed below.

Most studies with patients deal with a conventional sponge electrode setup. Anodal stimulation has been found to elicit cortical excitation over the area of activation. However, due to diffusion of the flow and different direction of the flow over the surface, different patterns of excitability may be occurring. Consequently, unwanted areas may be excited or inhibited [55]. In addition, the relative position between electrodes can vary the flow angle although not many studies have addressed this question thoroughly [91]. The relative position of the return electrode has been recognized as an important factor, and placing it in positions in the same hemisphere as the active electrode may produce a more specific and controlled effect [52].

Regarding the position of the electrodes and their effects, depending on the type of setup and electrode size, the maximum value of the electric field will vary in position. This is an important factor to take into account, which is not usually overemphasized. Smaller-sized electrodes have been more focal than sponge electrodes [52]. Cortical surface variations introduce variability, which is intensified with small electrodes and high intensities [94].

It has already been discussed which electrode positions are better and how the conventional setup can be improved. In the literature, the called conventional setup is the most applied, but it seems that the bilateral montage has shown good results in stroke patients. In stroke patients, anodal ipsilesional and cathodal stimulation localization is being used. However, no contralesional inhibition, only montage, has shown good results.

Anodal polarization is stimulated when the component is normal, but excitability has also been observed in different parts of the cell when the flow is tangent and showed promising results in areas of the hand [73]. In addition, it showed the best relationship in EF magnitude values and present low variability [94]. The anterior–posterior setup has not shown promising results in the leg area. Probably due to the homunculus leg position, the current flow patterns could be different from that of the upper limb [51].

Can HD stimulation improve these results? HD stimulation is used to target an area, all the advantages and disadvantages of which have been analyzed in this work with respect to the conventional setup. The most important factor is to increase the focality, the weak point of conventional tDCS. While the ring configuration has received the most attention in HD, details and variations produced by different numbers of return electrodes have also been addressed. Although there are no comparative studies of conventional and HD mounting on M1, some conclusions from the work done in simulations can be extracted. Regarding the 4 × 1 ring works, increasing the radius increases the magnitude of the field [76] and reduces the variability [94]. Cathodal polarity effects decrease with four return electrodes, comparable even when there are two [76]. In stroke patients, two works with HD configuration were found, both without much success, and only one was found with a lateral ring configuration [47]. In ring work in healthy subjects, the results were also unpromising [65,68].

HD stimulation allows for the stimulation or inhibition of two separate areas of the brain. If the relationship between the different areas were well known, this would allow for the creation of a map that, in a simplistic way, would activate and inhibit the network efficiently. However, there are studies that have investigated the relationships and effects that stimulation can have on SMA, M1 and somatosensory areas [71,103]. Only two studies showed promising results in the multiple active stimulation of non-local areas: the work that stimulates M1 and cerebellum in different pathways [70] and the work of [66] that stimulates DLPFC and motor area. However, the role of polarization is not as simple as this. In another view of tDCS as a noise modulator, cathodal tDCS can help to reduce noise in the circuit and bring about behavioral improvement under certain circumstances. Thus, understanding the tDCS process can explain the variability of some tasks [115]. The anodal polarization obtained worse results in the excitability of M1 than in the area of the hand [55], and the catodal did not obtain positive results in the M1 area [53].

Regarding the stimulation on M1, although there are surveys of positive results in the learning of a task, these are highly variable, which will be discussed later in detail. However, the efficacy of anodal stimulation for the performance of an ankle motor tracking task has been proved [56] so far in many of Madhavan’s et al. works, while in the cerebellum, stimulation also seems to obtain improvements in the learning of tasks when it is anodal [104] and especially when it is performed together with robot assistance [70].

Regarding the average stimulation time, whereas 15 min is usually normal, 20 min is usually applied. Shorter times call into question the effectiveness of the stimulation. Like time, intensity is not a parameter that has been excessively investigated. However, it must be considered that depending on the type of electrode and the number of return electrodes, the intensity should be chosen carefully as it could produce very diffuse effects.

Much of the variability observed in tDCS could come from the type of stimulation and task being performed. In single session studies, there is much variability in the conclusion of positive or negative results for most of the tested tasks. There is no clear pattern of stimulation (offline or online) combined with a task that effectively works in all task modalities.

However, many of Madhavan’s et al. work on tDCS+AMT reported positive results. The task during which the stimulation is performed may need to be similar to the one that is performed afterwards [66]. Performing online stimulation may not always have positive results, as has been commented in the protocols that involve tDCS+ robot assistance tasks [41,43,50].

It is possible that eliciting the plasticity effects of tDCS for a task is easier under certain conditions, due to the modulation that could be dependent on the activation pattern during stimulation [12] or under our assumption, perhaps on the task of validation or a combination of these.

Finally, recommendations are established as guidelines to follow in order to improve stroke and spinal cord injury protocols. In the case of stroke, the position of the stimulation is more decisive, due to neural damage and different incidences in the neural structure; individualizing protocols based on simulation software is a procedure that should gain strength. Moreover, adding a measure of brain activity to see how stimulation in certain positions affects the user population can give a measure of variability. Validating position with TMS or validating excitability is always recommended. However, if it is not possible to measure brain activity with EEG it should be a common practice. Having a measure of brain activity to be able to analyze and correlate it with behavior should be a more common practice. However, as mentioned, trying to find a common positioning protocol is important to the field. According to our vision, three types of strategy should be explored in more detail: first of all, the well-parameterized bilateral configuration between hemispheres, with enough distance between active and return [99], so that the current penetrates sufficiently (see Section 3.8). Secondly, protocols with greater focus and a better position centered in M1 should be implemented (see Section 3.5). Thirdly, the cerebellar M1 pathway has been successfully explored [70] and more research should be required to try to validate this protocol and its possible superiority over M1 stimulation, especially for the adaptation phase in motor tasks, especially in patients (where validation has already been attempted without much success [48]).

Switching to HD configurations, ring-4 × 1 configurations should continue to be explored, and they appear to be less than optimal. Although the HD configurations have not been shown to be much superior to the conventional ones or as the optimal paradigm, they obtain important advantages (see Section 3.6) over the conventional ones and should be explored in detail. With respect to spinal cord injury configurations, few studies have been reported, in this case, testing efficient M1 pathways [52], in which case symmetric configurations can be explored. Additionally, exploring the M1 pathway, cerebellum and spinal cord is a design pathway that looks promising [110].

In addition, it must be taken into account under which conditions to apply these configurations. i.e., whether to parameterize online or offline tDCS according to the evidence on the task to be implemented. Electrode positions and configurations are also correlated with the task and the objective to be achieved, and reducing variability and increasing reproducibility should be the goal of the field.

Analyzing the works separately has allowed us to find weaknesses and all the characteristics to take into account when setting up a better protocol for the stimulation of the lower limb. The next step is to propose an excitability configuration in M1 that will be considered as a standard model of HD stimulation.

## 5. Conclusions

In this work, an in-depth review of the work on tDCS for the lower-limb has been carried out. The objective is to create more stable and effective protocols for rehabilitation therapies that, together with novel rehabilitation mechanisms (tDCS, BCI and exoskeletons) may accelerate and improve rehabilitation processes. The premise is to find a configuration that presents less variability than the conventional setup, due to the fact that individualizing all the stimulation protocols by MRI is costly and impractical. With the conclusions drawn in this work, the first step for future work is to compare simulated models to observe the variability and penetrability of the EF on the brain and target area. Later, in a control group study, it will be observed whether the proposed protocol produces changes at the level of neural connectivity and efficacy in the improvement of an exoskeleton control task by means of BCIs.

## Figures and Tables

**Table 1:** Stroke.

	Users	Location		Intensity/Current Density		Session	Task	Time	Groups	Measures	Outcomes
	(Type)	Anode	Cathode	Anode	Cathode	Numbers		tDCS (min)		Intervals	Measures
[29]	9 chronic AG	8 cm${}^{2}$ ILe M1 leg	48 cm${}^{2}$ CLe SOr	0.5 mA 0.0625 mA/cm${}^{2}$	0.5 mA 0.0104 mA/cm${}^{2}$	3 (2 tDCS)	Online/AMT	15	before/after 48 h between sessions	(Sham/anodal ipsilesional tDCS/anodal contralesional tDCS+AMT)	(+)MEP
[30]	11 chronic GD	8 cm${}^{2}$ ILe M1 leg	35 cm${}^{2}$ CLe Sor	1 mA 0.125 mA/cm${}^{2}$	1 mA 0.028 mA/cm${}^{2}$	2 (1 tDCS)	Offline but visual tracking task/TT session	10	before/after 1 week between sessions	(Sham/tDCS+TT)	(+)MEP GS
[31]	12 subacute GD	sponge 7.07 cm${}^{2}$ ILe M1 leg (TMS Hspt TA)	sponge 28.26 cm${}^{2}$ CLe SOr	2 mA 0.28 mA/cm${}^{2}$	2 mA 0.07 mA/cm${}^{2}$	10 c	Online/CPT	10	1 day before/1 day after 2 weeks 2 days between sessions	(Sham + CPT) (tDCS+CPT)	(+)MEP (+)FMA, (+)IM, FAC, BBS
[32]	18 chronic GD	25 cm${}^{2}$ ILe M1 leg (TMS Hspt quadriceps)	25 cm${}^{2}$ CLe SOr	2 mA 0.08 mA/cm${}^{2}$	2 mA 0.08 mA/cm${}^{2}$	2 (1 tDCS)	Online/ Evaluation test	20	1 day before/during/1 h 10 days between sessions	(Sham/tDCS+evaluation test)	(+)6MWT
[33]	13 chronic AG	35 cm${}^{2}$ C3 or C4 ILe ILe Sor	35 cm${}^{2}$ CLe SOr C3 or C4 Cle	2 mA 0.057 mA/cm${}^{2}$	2 mA 0.057 mA/cm${}^{2}$	3 (2 tDCS)	Offline/TA task realted lower-limb task	15	before/after 1 week between sessions	(a-tDCS/c-tDCS/Sham)	(-)FMA, IM BBS
[34]	8 chronic GD	35 cm${}^{2}$ ILe M1 leg	50 cm${}^{2}$ CLe SOr	2 mA 0.057 mA/cm${}^{2}$	2 mA 0.04 mA/cm${}^{2}$	2 (1 tDCS)	Online/Maximum force knee extension	10	before/during/ 30 min	(tDCS) (Sham)	(+)MF
[35]	19 subacute AG	35 cm${}^{2}$ ILe M1 leg	35 cm${}^{2}$ CLe M1 leg	2 mA 0.057 mA/cm${}^{2}$	2 mA 0.057 mA/cm${}^{2}$	2 (1 tDCS)	Offline/CPT	20	before/after 1 week between sessions	(Sham/tDCS)	(+)FTSTS, MS
[36]	10 chronic AG	35 cm${}^{2}$ ILe C3 or C4	35 cm${}^{2}$ CLe C3 or C4	2 mA 0.057 mA/cm${}^{2}$	2 mA 0.057 mA/cm${}^{2}$	2 (1 tDCS)	Offline/gross motor task	20	before/after	(Sham/tDCS)	PT, (+)FS
[37]	16 subacute AG 15 subacute AG	35 cm${}^{2}$ C4 or C3 Ile	35 cm${}^{2}$ C4 or C3 Cle	1.5 mA 0.04 mA/cm${}^{2}$	1.5 mA 0.04 mA/cm${}^{2}$	32 (16 s tDCS)	None	20	before/after 4 weeks 1 week between sessions	(Sham/tDCS+evaluation test) (tDCS/Sham +evaluation test)	(+)Tinetti, RMA, TIS
[38]	21 chronic GD	25 cm${}^{2}$ ILe M1 leg (TMS Hspt hallucis muscle)	25 cm${}^{2}$ CLe SOr	2 mA 0.08 mA/cm${}^{2}$	2 mA 0.08 mA/cm${}^{2}$	10 c	Offline/RAGT	20	before/after/4 weeks	(Sham) (tDCS)	(+)FAC, 10MWT, 6MWT, FMA BBS MEP
[39]	10 chronic GD	25 cm${}^{2}$ Cz	35 cm${}^{2}$ F SOr	2 mA 0.08 mA/cm${}^{2}$	2 mA 0.057 mA/cm${}^{2}$	12 s	Offline/RGO	20	before/after 4 weeks/1 month	(Sham) (tDCS)	(+)FAC, (+)SIS, 10MWT, (+)TUG BBS
[40]	24 chronic GD	not specified Cz L area	not specified R SOr	2 mA	2 mA	12 s	Online/Task related training	15	before/after 4 weeks	(TRT) (Sham tDCS+TRT) (tDCS+TRT)	TWP
[41]	32 subacute GD	35 cm${}^{2}$ Cz	35 cm${}^{2}$ R SOr	2 mA 0.057 mA/cm${}^{2}$	2 mA 0.057 mA/cm${}^{2}$	20 c	Online during first 20 min/RAGT	20	before/after 4 weeks	(Sham+RAGT) (tDCS+RAGT)	GS FAC
[42]	76 chronic (GD)	12.5 cm${}^{2}$ ILe M1 leg (TMS Hspt TA)	24.75 cm${}^{2}$ CLe Sor	1 mA 0.08 mA/cm${}^{2}$	1 mA 0.04 mA/cm${}^{2}$	12 s	Offline but one group Online perform AMT/TT	15	before/after 4 weeks/3 months	(Control) (tDCS) (AMT) (tDCS+AMT)	(+)MEP GS
[43]	30 chronic GD	35 cm${}^{2}$ ILe M1 leg	35 cm${}^{2}$ CLe SOr	1.5 mA 0.04 mA/cm${}^{2}$	1.5 mA 0.04 mA/cm${}^{2}$	10 c	Online during the first 7 min/RAGT	7	before/after 2 weeks/2 weeks	(Sham+RAGT) (Sham+RAGT) (walking exercises)	6MWT, 10MWT
[44]	60 acute GD	35 cm${}^{2}$ C3 or C4 ILe ILe SOr C3 or C4 Ile	35 cm${}^{2}$ CLe SOr C3 or C4 CLe C3 or C4 Cle	2 mA 0.05 mA/cm${}^{2}$	2 mA 0.05 mA/cm${}^{2}$	10 c	None	Not specified	before/after 2 weeks/1 and 3 months	(a ipsilesional-tDCS) (c contralesional-tDCS) (bilateral) (Sham)	(+)FSST, 6MWT (+)OSI, OFI, FES, BBS SST
[45]	14 subactue GD	25 cm${}^{2}$ ILe M1 leg	25 cm${}^{2}$ CLe M1 leg	2 mA 0.08 mA/cm${}^{2}$	2 mA 0.08 mA/cm${}^{2}$	1	None	15	not specified	(Sham) (tDCS)	(+)TUG POMA
[46]	16 chronic AG	1.75 cm${}^{2}$ M ILe Rf CLe shoulder DM ILe M1 leg Rf on the ILe shoulder	Rf on the IL shoulder M CLe DM CLe M1 leg Rf on the CLe shoulder	0.175 mA 0.1 mA/cm${}^{2}$ 0.175 mA 0.1 mA/cm${}^{2}$	0.175 mA 0.1 mA/cm${}^{2}$ 0.175 mA 0.1 mA/cm${}^{2}$	4 (3 tDCS)	Online/TT	20	before/after 48 h between sessions	(Sham/a-tDCS/c-tDCS/bilateral tDCS+TT)	FMA-LE BBS GI, GS PSR
[47]	18 chronic AG	HD-4.5 cm${}^{2}$ C2 or C1 ILe (TMS Hspt TA) F2, F4, Pz, P4 or Fz, F3, Pz, P3	HD-4.5 cm${}^{2}$ F2, F4, Pz, P4 or Fz, F3, Pz, P3 C2 or C1 ILe (TMS Hspt TA)	2 mA 4.52 mA/cm${}^{2}$ 0.5 mA 0.11 mA/cm${}^{2}$	0.5 mA 0.11 mA/cm${}^{2}$ 2 mA 4.52 mA/cm${}^{2}$	3 (1 tDCS)	Online/Pedaling	20	before/after not specified	(Sham/tDCS +evaluation test)	MEP BBS, FGA, 6MWT, FMA
[48]	6 subacute GD	HD-3.14 cm${}^{2}$/HD 3.14 cm${}^{2}$ Cz/2 cm R or L CLe In	HD-3.14 cm${}^{2}$ FC2 or FC1 Cle	0.2 mA/0.3 mA 0.06 mA/cm${}^{2}$/ 0.09 mA/cm${}^{2}$	0.5 mA 0.16 mA/cm${}^{2}$	5 c	Offline/MI and pedaling exercise	15	before/during/ 2 months	Sham tDCS	BipHS FAC, MRS, IM, FDS MI-FC

**Table 2:** SCI.

	Users	Location		Intensity/Current Density		Session	Task	Time	Groups	Measures	Outcomes
	(Type)	Anode	Cathode	Anode	Cathode	Numbers		tDCS (min)		Intervals	Measures
[49]	15 C GD	25 cm${}^{2}$ Cz ( or TMS Hspt LE)	35 cm${}^{2}$ F Sor	2 mA 0.08 mA/cm${}^{2}$	2 mA 0.06 mA/cm${}^{2}$	36 s	Offline/RGO	20	(Sham+RGO) (tDCS+RGO)	before/after 12 weeks/1 month	(+)MMT 6MWT, 10MWT, TUG, BBS (-)SCIM-III
[50]	24 C GD	35 cm${}^{2}$ Cz	35 cm${}^{2}$ non-dominant leg Sor	2 mA 0.06 mA/cm${}^{2}$	2 mA 0.06 mA/cm${}^{2}$	20 c	Online/RGO	20	(Sham+RGO) (tDCS+RGO)	before/after 4 weeks/1 month	MS, GI, 10MWT

**Table 3:** Healthy.

	Users	Location		Intensity/Current Density		Session	Task	Time	Groups	Measures	Outcomes
	(Type)	Anode	Cathode	Anode	Cathode	Numbers		tDCS (min)		Intervals	Measures
[51]	20 AG	13 cm${}^{2}$ TA (hotspot measured with TMS) C3 or C4 5cm posterior	35 cm${}^{2}$ CLe SOr C3 or C4 5cm anterior	1 mA 0.076 mA/cm${}^{2}$	1 mA 0.028 mA/cm${}^{2}$ 0.076 mA/cm${}^{2}$	2 (2 tDCS)	Online/AMT	15	(tDCS+conventional/ tDCS+anterio-posterior)	before/after 7 days between sessions	MEP
[52]	10 AG	35 cm${}^{2}$ M1 R leg (Hspt TMS) 3.5 cm${}^{2}$ M1 R leg (Hspt TMS) 3.5 cm${}^{2}$ M1 R leg (Hspt TMS)	35 cm${}^{2}$ Fp2 Fp2 T7	1 mA 0.028 mA/cm${}^{2}$ 0.2 mA 0.057 mA/cm${}^{2}$ 0.2 mA 0.057 mA/cm${}^{2}$	1 mA 0.028 mA/cm${}^{2}$ 1 mA 0.028 mA/cm${}^{2}$ 1 mA 0.028 mA/cm${}^{2}$	4 (3 tDCS)	None	10	(tDCS setup1/tDCS setup2/tDCS setup3/Sham)	before/after 1 week between sessions	MEP (target hotspot, CLa hotspot, (+)Specifity)
[53]	8 AG	35 cm${}^{2}$ M1 L leg (TMS Hspt TA) CLa SOr	35 cm${}^{2}$ CLa SOr M1 L leg (TMS Hspt TA)	2 mA 0.06 mA/cm${}^{2}$	2 mA 0.06 mA/cm${}^{2}$	3 (2 tdcs)	Online or Offline/not specified	10	(a-tDCS/c-tDCS/Sham)	before/after 3 days between sessions	(+)MEP
[13]	12 GD	25 cm${}^{2}$ M1 3cm R I (TMS Hspt TA)	45 cm${}^{2}$ R buccinator muscle	2 mA 0.08 mA/cm${}^{2}$	2 mA 0.0044 mA/cm${}^{2}$	5 (2tDCS)	Online/differents MI states	15	(Sham/MIs-tDCS/MIc-tDCS)	before/after 1 week between senssions	MEP
[54]	15 AG	12.5 cm${}^{2}$ non-dominant M1 leg (Hspt TMS TA)	35 cm${}^{2}$ dominant SOr	1 mA 0.08 mA/cm${}^{2}$	1 mA 0.03 mA/cm${}^{2}$	4 (4 tDCS)	Online/Skilled motor task or Offline	15	(tDCS+skilled motor task/tDCS+rest)	after, before, 10 min and 30 min 7 days between sessions	MEP
[55]	10 AG	8 cm${}^{2}$ Cz 1 cm L or Cz 1 cm R	48 cm${}^{2}$ CLa SOr	0.5 mA 0.06 mA/cm${}^{2}$	0.5 mA 0.01 mA/cm${}^{2}$	4 (4 tDCS)	Offline/skilled motor task	10	(tDCS-left/tDCS-right)	before/after several days	MEP
[56]	12 AG	8 cm${}^{2}$ M1 leg (TMS hotspot TA)	35 cm${}^{2}$ CLa SOr	1 mA 0.13 mA/cm${}^{2}$	1 mA 0.028 mA/cm${}^{2}$	3 (2 tDCS)	Online or Offline/AMT	15	(Sham/tDCS+AMT/tDCS)	before/during/after/ 10 min, 25 min and 24 h 7 days between sessions	(+)MEP MMT
[57]	14 AG	sponge 25 cm${}^{2}$ Cz	35 cm${}^{2}$ F SOr	1 mA 0.04 mA/cm${}^{2}$	1 mA 0.03 mA/cm${}^{2}$	3 (2 tDCS)	Online or Offline/MI training	10	(Control/Sham+MI /tDCS+MI)	before/after 1 week between sessions	(+)BBS
[58]	30 GD	25 cm${}^{2}$ 1 cm behind Cz	50 cm${}^{2}$ R SOr	1 mA 0.04 mA/cm${}^{2}$	1 mA 0.02 mA/cm${}^{2}$	2 (1 tDCS)	Online only during the first session/dynamic balance task	20	(tDCS) (Sham)	before/during	TiB
[59]	14 AG	12.5 cm${}^{2}$ non-dominant M1 TA	35 cm${}^{2}$ CLa SOr	1 mA 0.08 mA/cm${}^{2}$	1 mA 0.03 mA/cm${}^{2}$	2 (1 tDCS)	Online/Skilled motor task	15	(Sham/tDCS+skilled motor task)	before/during 7 days between sessions	Ankle movility WE, (+)SRT and (+)CRT SDMT
[60]	16 AG	25 cm${}^{2}$ Cz	35 cm${}^{2}$ F SOr	1 mA 0.04 mA/cm${}^{2}$	1 mA 0.03 mA/cm${}^{2}$	3 (3 tDCS)	Online or Offline/MI training	10	(tDCS before/tDCS during/tDCS before+during)	before/after 1 week between sessions	(+)BBS
[61]	32 GD	55.25 cm${}^{2}$ M1 dominant leg	55.25 cm${}^{2}$ CLa SOr	1 mA 0.02 mA/cm${}^{2}$	1 mA 0.02 mA/cm${}^{2}$	5 c	None	20	(tDCS/Sham)	before/after 1 week/1 week	(+)TUG (+)30 s CST (+)MFEW
[62]	14 AG		35 cm${}^{2}$ M R inion Rf ILa houlder		2 mA 0.057 mA/cm${}^{2}$	2 (1 tDCS)	Offline/Counter balancing task	20	(tDCS/sham)	before/during 1 week between sessions	Task acuraccy, CRT
[13]	12 AG	25 cm${}^{2}$ M R inion Rf bucinator muscle		2 mA 0.08 mA/cm${}^{2}$	2 mA 0.044 mA/cm${}^{2}$	2 (1 tDCS)	Offline/MI task	20	(tDCS/sham)	before/after 1 week between sessions	(+)MEP
[63]	40 GD	25 cm${}^{2}$ 3 cm R or L In Rf Ila buccinator	25 cm${}^{2}$ 3 cm R or L In Rf Ila buccinator	2 mA 0.08 mA/cm${}^{2}$	2 mA 0.08 mA/cm${}^{2}$	1	Offline but tDCS are supply during adaptation/Walking adaptation TT	20	(Sham) (a-tDCS) (c-tDCS)	before/during/after	(+)Walking simetry parameters
[64]	24 GD	25 cm${}^{2}$ M M1 non-dominant leg Rf on the ILa upper arm		2 mA 0.04 mA/cm${}^{2}$		7 s	Online/LE muscle strength	10	(Sham+LE muscle strength) (tDCS+LE muscle strength)	before/after 3 weeks	MF
[65]	14 GD	HD-0.78 cm${}^{2}$ Cz	HD-0.78 cm${}^{2}$ FCz/C3/Pz/C4	2 mA 2.54 mA/cm${}^{2}$	2 mA 2.54 mA/cm${}^{2}$	2 (1 tDCS)	None	20	(Sham) (tDCS)	before/after	(+)MF
[66]	27 AG	HD-3.14 cm${}^{2}$ Cz/F3	HD-3.14 cm${}^{2}$ FC5/FC1/AF4/CP1	1.5 mA/1.5 mA 0.47 mA/cm${}^{2}$/0.47 mA/cm${}^{2}$	0.75 mA/0.96 mA/0.53 mA/0.75 mA 0.23 mA/cm${}^{2}$/0.3 mA/cm${}^{2}$/0.16 mA/cm${}^{2}$/0.23 mA/cm${}^{2}$	1+3(2)	Online/TT	20	(tDCS+seated /tDCS+walking /Sham+walking)	before/after 3 days between sessions	(+)GS Stroop Color, Word Test, SDMT
[67] [68]	5 AG 5 AG	HD-3.14 cm${}^{2}$ between Cz and FC1 HD-3.14 cm${}^{2}$ Cz	HD-3.14 cm${}^{2}$ 2 cm L In HD-3.14 cm${}^{2}$ FC1/FC2/CP1/CP2	0 mA 0.06 mA 0.02 mA/cm${}^{2}$ 0.125 mA 0.04 mA/cm${}^{2}$ 0.18 mA 0.06 mA/cm${}^{2}$	0 mA 0.06 mA 0.02 mA/cm${}^{2}$ 0.125 mA 0.04 mA/cm${}^{2}$ 0.18 mA 0.06 mA/cm${}^{2}$	1	Offline/MI walking	10	(tDCS)	after	MI-FC
[69]	14 GD	HD-3.14 cm${}^{2}$ Cz	HD-3.14 cm${}^{2}$ 2 cm R In	0.4 mA 0.13 mA/cm${}^{2}$	0.4 mA 0.13 mA/cm${}^{2}$	5 c	Offline/MI walking	15	(Sham) (tDCS)	after	(+)MI-FC
[70] [71]	12 GD 4 GD	HD-3.14 cm${}^{2}$ Cz/2 cm R In	HD-3.14 cm${}^{2}$ FC2	0.2 mA/0.3 mA 0.06 mA/cm${}^{2}$/0.09 mA/cm${}^{2}$	0.5 mA 0.16 mA/cm${}^{2}$	5 c	Offline/MI walking Close-loop Offline/MI walking with exoskeleton Close-loop	15	(Sham) (tDCS)	after	(+)MI-FC

## Data Availability

Not applicable.

## Abbreviations

The following abbreviations are used in this manuscript:

30sCST	30s Chair Stand Test
MF	Maximum Force
AG	All groups
MFEW	Modified Figure of Eight Walk
AMT	Ankle motor tracking
MI	FC-Motor imageri feature classification
BBS	Berg Balance Scale
MMT	manual muscle testing
BipHS	Standing of the Hospital of Sagunto
MRS	Modified Ranking Scale
CLa	Contralateral
MS	motor score
CLe	Contralesional
MWT	Min Walking Test
CPT	Conventional physical therapy
OFI	Occurrence of Falling Index
CRT	choice reaction time
OSI	Overall Stability Index
DBT	Dynamic balance task
PE	Pedaling exercise
EF	ElectricField
POMA	Performance Oriented Mobility Assessment
F	Frontal
PSR	Paretic Step Ratio
FAC	Functional Ambulatory Category
PT	peak muscular torque
FDS	Force of Daniels Scale
R	Right
FES	Falls Efficacy Scale
RAGT	Robot asistance gait training
FGA	Functional Gait Assessment
Rf	Reference
FMA	Fugl Meyer Assessment
RGO	Robot gait orthesis
FS	Force steadiness
RMA	Rivermead Motor Assessment
FTSTS	Five Times Sit To Stand
SCIM-III	Spinal Cord Independence Measure-III
GD	Group division
SDMT	symbol digit modality test
GI	Gait Index
SMT	Skilled Motor Task
GMT	Gross motor task
SOr	Supra orbital
GS	Gait Speed
SRT	simple reaction time
Hspt	Hospot
SST	Sit to Stand Test
ILa	Ipsilateral
TA	Tibialis Anterioris
ILe	Ipsilesional
TiB	Time in Balance
IM	Index Motricity
TIS	Trunk Impairment Scale
IM	Index motor
TRT	Task related training
In	Inion
TT	Treadmill training
L	Left
TUG	Time Up and Go Test
M	monopolar
TWP	Temporal Walking Parameters
MEP	Motor Evoked Potential
SIS	Stroke Impact Scale
